# 

**Published:** 1894-09

**Authors:** 


### UNITED STATES VETERINARY MEDICAL ASSOCIATION.

The United States Veterinary Medical Association will hold their Thirty-first Annual Meeting at the Academy of Natural Science, 19th and Race Streets, Philadelphia, Pa., September 18th, 19th and 20th, 1894.

All railroads in the territory east of Chicago have granted the usual 1⅓ rates.

Hotel Hanover, 12th and Arch Streets, will be the headquarters of the members

The afternoon and evening of the 17th will be taken up by a meeting of the Association of Veterinary Faculties of the Colleges of North America, in conjunction with the Committee on Congress of Colleges, at 3 P. M.; and meeting of the Comitia Minora at 8 P. M., for the discussion of preliminary business and consideration of applications for membership. Meetings on the 17th will be held at Hotel Hanover.

A meeting of the Comitia Minora will be held each morning at 9 A. M.

The Convention will be welcomed on the 18th by an address by Mayor Edwin S. Stuart, at 10 A. M., which will be followed by the President's address. The remainder of the day will be consumed by the consideration of the report of the Comitia Minora and various other committees.

The meeting on the 19th will convene with roll call at 10 A.M., after which the report of the Comitia Minora, and reports of Special Committees on Congress of Colleges, Act of Incorporation, Revision of Constitution and By-laws, and Nomenclature of Swine Plague and Hog Cholera, followed by the election of officers and any other new business. The afternoon will be given to reading of papers on "Neurotomy as a Practical Operation," by Dr. S. J. J. Harger, of Philadelphia, Pa., and "Psychology of the Domestic Animals," by Dr. Olaf Schwarzkopf, Chicago, Ill.; also, unfinished discussion of paper on "A New Method of Treating Periodic Ophthalmia by Surgical Interference."

The meeting on the 20th will be consumed by reading of papers on "Tuberculosis," by Drs. J. Faust, Poughkeepsie, N. Y., M. R. Trumbower, Sterling, Ill., and Leonard Pearson, Philadelphia, Pa.; "The Peculiarities of the Diseases of the Rocky Mountain Regions," by Dr. W. L. Williams, Bozeman, Mont.; and

"Horseshoeing," by Dr. L. McLean, Brooklyn, N. Y., followed by discussion of said papers.

The Zoological Society has extended to the visiting members a request that they visit the gardens here in a body.

The Convention will close with a banquet on the evening of the 20th.

The Local Committee of Arrangements recommend the following hotels as convenient and well adapted for entertaining all who may attend: Hotel Hanover, 12th and Arch Streets, $2.50 per day and upwards; Bingham House, 11th and Market Streets, $2.50 per day and upwards; Hotel Lafayette, Broad and Chestnut Streets, $3 per day and upwards; Colonnade Hotel, corner of 15th and Chestnut Streets, European plan, $2; American plan, $3 and upwards.

